# Home Repair Policies and the Uneven Landscape of Aging in Community for Low-Income Older Adult Homeowners

**DOI:** 10.1093/geroni/igaf122.3789

**Published:** 2025-12-31

**Authors:** Leiha Edmonds

**Affiliations:** Indiana University Bloomington, Bloomington, Indiana, United States

## Abstract

Federal, state, and local policies in the United States are increasingly recognizing home repair as a strategy for supporting age-friendly cities and communities. Ensuring access to home maintenance, repairs, and modifications is crucial to support aging in place, enhance the physical and mental health of older adults, and enable low-income homeowners to resist displacement pressures. This article investigates the influence of housing policy on aging-in-place experiences among low-income older adults by examining the work of the Orange County Home Preservation Coalition (OCHPC) in the Triangle Region of North Carolina. The OCHPC comprises local residents, municipal governments, nonprofit organizations, and home repair providers who collaboratively deliver free home improvements to older adults with limited incomes. Using policy analysis, administrative and interview data from coalition partners, we assess how federal, state, and local policies shape the implementation and reach of these repair programs. Our findings indicate that, despite strong rhetorical commitments to affordable housing stability and climate resilience for older adults, geographic eligibility criteria embedded in policy frameworks result in uneven access to home repair services, particularly disadvantaging those living in peri-urban areas (regions adjacent to metropolitan centers). We further explore how collaborative, cross-sector coalitions can leverage and integrate diverse funding streams and programs to more comprehensively address the home repair needs of low-income older adults.

